# Streptococcal toxic-shock syndrome: spectrum of disease, pathogenesis, and new concepts in treatment.

**DOI:** 10.3201/eid0103.950301

**Published:** 1995

**Authors:** D L Stevens

**Affiliations:** University of Washington School of Medicine, Seattle, Washington, USA.

## Abstract

Since the 1980s there has been a marked increase in the recognition and reporting of highly invasive group A streptococcal infections with or without necrotizing fasciitis associated with shock and organ failure. Such dramatic cases have been defined as streptococcal toxic-shock syndrome. Strains of group A streptococci isolated from patients with invasive disease have been predominantly M types 1 and 3 that produce pyrogenic exotoxin A or B or both. In this paper, the clinical and demographic features of streptococcal bacteremia, myositis, and necrotizing fasciitis are presented and compared to those of streptococcal toxic-shock syndrome. Current concepts in the pathogenesis of invasive streptococcal infection are also presented, with emphasis on the interaction between group A Streptococcus virulence factors and host defense mechanisms. Finally, new concepts in the treatment of streptococcal toxic-shock syndrome are discussed.


### Streptococcal Toxic-Shock Syndrome: Spectrum of Disease, Pathogenesis, and New Concepts in Treatment

**Dennis L. Stevens, Ph.D., M.D.**
Professor of Medicine, University of Washington School of Medicine, Seattle, Washington
Chief, Infectious Disease Section, Veterans Affairs Medical Center, Boise, Idaho

*Since the 1980s there has been a marked increase in the recognition and reporting of highly invasive group A streptococcal infections with or without necrotizing fasciitis associated with shock and organ failure. Such dramatic cases have been defined as streptococcal toxic-shock syndrome. Strains of group A streptococci isolated from patients with invasive disease have been predominantly M types 1 and 3 that produce pyrogenic exotoxin A or B or both. In this paper, the clinical and demographic features of streptococcal bacteremia, myositis, and necrotizing fasciitis are presented and compared to those of streptococcal toxic-shock syndrome. Current concepts in the pathogenesis of invasive streptococcal infection are also presented, with emphasis on the interaction between group A* Streptococcus *virulence factors and host defense mechanisms. Finally, new concepts in the treatment of streptococcal toxic-shock syndrome are discussed.*

An emerging pathogen can be one that is totally new (e.g., human immunodeficiency virus), one that was known but has only recently been identified (e.g., *Helicobacter pylori*), or one that is old but has learned new tricks. The last type is, as Dr. Stanley Falkow contends, merely trying to "make a living" in a changing environment. Regardless of environmental pressures, many old pathogens have become major clinical problems because of increased virulence or antibiotic resistance (e.g., penicillin-resistant pneumococcus, multidrug resistant *Mycobacterium tuberculosis*, methicillin-resistant *Staphylococcus aureus*, and vancomycin-resistant *Enterococcus faecium*).

Arguably, group A *Streptococcus* (GAS) is the quintessence of an old organism that has become more virulent. In this manuscript, the epidemiology, clinical spectrum, and pathogenesis of GAS infection are discussed in relation to the streptococcal toxic-shock syndrome (TSS).

### Current and Historical Perspectives on the Prevalence and Severity of Streptococcal Infections

The British tabloids have recently coined the term "flesh-eating bacteria" to describe invasive necrotizing infections caused by GAS and have suggested that epidemics of streptococcal infection are imminent. Such aggrandizement is unfounded, yet it has served to heighten public awareness of this sporadic, but serious, infectious disease. Strictly speaking, an epidemic is defined as an increase in the prevalence of disease over a baseline endemic rate. In this context, we are, in fact, experiencing an epidemic of severe invasive GAS infections; however, few concrete prospective population-based data support this notion. Estimates suggest that the incidence of these infections is 10 to 20 cases/ 100,000 population. Thus, the stimulus for such public interest has not been the incidence of the syndrome, but more likely, the dramatic nature of these infections.

Whether these types of group A streptococcal infections will decline, stay the same, or increase is not known. History is replete with descriptions of epidemics of GAS infections and their nonsuppurative sequelae. In the 1600s, epidemics of scarlet fever spread from Italy and Spain to Northern Europe (1), and in 1736, an outbreak occurred in the American colonies, killing 4,000 people (2). Major epidemics of rheumatic fever occurred in World War II in the U.S. military (3). Soon afterward post-streptococcal glomerulonephritis struck several regions of the United States (4,5).

Many of these epidemics waxed and waned before the advent of antibiotics, suggesting that either changes in socioeconomic conditions or variations in the expression of virulence factors by the pathogen were responsible. This concept is best exemplified by the extraordinary mortality rate of scarlet fever documented in the latter part of the 1880s in New York, Chicago, and Norway; 25% to 30% of children with scarlet fever died during that period (5,6). By 1900, the mortality rate had dropped to under 2% in all three locations. Since socioeconomic conditions

Address for correspondence: Infectious Disease Section, Veterans Affairs Medical Center, 500 West Fort Street (Bldg 6), Boise, ID 83702, USA; fax: 208-389-7965.

likely did not change markedly during that time and antibiotics were not yet available, the decrease in mortality rates must have been caused by reduced expression of a streptococcal virulence factor or by the slow acquisition of herd immunity to that factor.

The epidemiology of GAS infection is complex. More than 80 different M types of *S. pyogenes* exist, and five separate and distinct scarlatina toxins, streptococcal pyrogenic exotoxins (SPEs) (5) have also been described; some of these can be transmitted to different M types by bacteriophage. Minor drifts in the antigenic or virulence properties of GAS could account for the 5- to 6-year cycles of scarlet fever documented by Kohler (9). In the same way as antigenic shifts in influenza virus cause pandemics, major alterations in GAS virulence properties could cause major changes in clinical disease. The recent increases in severe GAS infections, following a 50- to 60-year span of relatively benign clinical disease, support this notion.

### Acute Life-Threatening Group A Streptococcal Infections

### Streptococcal TSS

Recently, severe invasive GAS infections associated with shock and organ failure have been reported with increasing frequency, predominantly from North America and Europe (8-18). These infections have been termed streptococcal toxic-shock syndrome (TSS; Table 1) (19). Persons of all ages are affected; most do not have predisposing underlying diseases (11,20-25). This is in sharp contrast to previous reports of GAS bacteremia, in which patients were either under 10 or over 60 years of age, and most had underlying conditions such as cancer, renal failure, leukemia, or severe burns or were receiving corticosteroids or other immunosuppressing drugs (20-22). The complications of current GAS infections are severe; bacteremia associated with aggressive soft tissue infection, shock, adult respiratory distress syndrome and renal failure are common; 30% to 70% of patients die in spite of aggressive modern treatments (Table 2) (1,8,24-26).

### Acquisition of Group A *Streptococcus*

The portal of entry of streptococci cannot be proven in at least half the cases (8) and can only be presumed in many others. Patients with symptomatic pharyngitis rarely develop streptococcal TSS, though such cases have been reported, especially in the last year. Procedures such as suction lipectomy, hysterectomy, vaginal delivery, bunionectomy and bone pinning have provided a portal of entry in many cases (author's unpublished observations). Most commonly, infection begins at a site of minor local trauma, which frequently does not result in a break in the skin (8). Numerous cases have developed within 24 to 72 hours of minor nonpenetrating trauma, resulting in hematoma, deep bruise to the calf, or even muscle strain. Virus infections, such as varicella and influenza, have provided a portal in other cases. In some cases the use of nonsteroidal antiinflammatory agents may have either masked the early symptoms or predisposed the patient to more severe streptococcal infection and shock (1). For the most part, these infections have occurred sporadically and have not been associated with clusters of cases or minor epidemics, though outbreaks of severe GAS infections have occurred in closed environments such as nursing homes (27,28).

### Clinical Symptoms

Pain—the most common initial symptom of streptococcal TSS—is abrupt in onset and severe, and usually precedes tenderness or physical findings. The pain usually involves an extremity but may also mimic peritonitis, pelvic inflammatory disease, pneumonia, acute myocardial infarction, or pericarditis. Twenty percent of patients have an influenza-like syndrome characterized by fever, chills, myalgia, nausea, vomiting, and diarrhea (8).

Fever is the most common early sign, although hypothermia may be present in patients with shock. Confusion is present in 55% of patients, and in some, coma or combativeness is manifest (8). Eighty percent of patients have clinical signs of soft tissue infection, such as localized swelling and erythema, which in 70% of patients progressed to necrotizing fasciitis or myositis and required surgical debridement, fasciotomy or amputation (8). An ominous sign is the progression of soft tissue swelling to the formation of vesicles, then bullae, which appear violaceous or bluish. In such patients, emergent surgical exploration should be performed to establish the diagnosis and distinguish GAS infection from other necrotizing soft tissue infections. Among the 20% of patients without soft tissue findings, clinical symptoms include endophthalmitis, myositis, perihepatitis, peritonitis, myocarditis, and overwhelming sepsis. A diffuse, scarlatina-like erythema occurs in only 10% of patients. Nearly 50% of patients may have normal blood pressure (systolic pressure >110 mm Hg) on admission but develop hypotension within the subsequent 4 hours (8).

### Laboratory Evaluation of Patients

On admission, renal involvement is indicated by the presence of hemoglobinuria and by serum creatinine values that are, on average, >2.5 times normal. Renal impairment precedes hypotension in 40% to 50% of patients (8). Hypoalbuminemia is associated with hypocalcemia on admission and throughout the hospital course. The serum creatinine kinase level is useful in detecting deeper soft-tissue infections; when the level is elevated or rising, there is a good correlation with necrotizing fasciitis or myositis. Though the initial laboratory studies demonstrate only mild leukocytosis, the mean percentage of immature neutrophils (including band forms, metamyelocytes, and myelocytes) is striking, reaching 40% to 50%. Blood cultures are positive in 60% of cases (8).

### Clinical Course

Shock is apparent at the time of admission or within 4 to 8 hours in virtually all patients (Table 2). In only 10% of patients does systolic blood pressure become normal 4 to 8 hours after administration of antibiotics, albumin, and electrolyte solutions containing salts or dopamine; in all other patients, shock persists. Similarly, renal dysfunction progresses or persists in all patients for 48 to 72 hours in spite of treatment, and many patients may require dialysis (8). In patients who survive, serum creatinine values return to normal within 4 to 6 weeks. Renal dysfunction precedes shock in many patients and is apparent early in the course of shock in all others. Acute respiratory distress syndrome

**Table 1. Case definition of streptococcal toxic-shock syndrome (streptococcal TSS) and necrotizing fasciitis***

I. Streptococcal TSS
- A. Isolation of group A *Streptococcus*
  - 1. From a sterile site
  - 2. From a nonsterile body site
- B. Clinical signs of severity
  - 1. Hypotension
  - 2. Clinical and laboratory abnormalities (requires two or more of the following):
    - a) Renal impairment
    - b) Coagulopathy
    - c) Liver abnormalities
    - d) Acute respiratory distress syndrome
    - e) Extensive tissue necrosis, i.e., necrotizing fasciitis
    - f) Erythematous rash

Definite Case = A1 + B(1+2)
Probable Case = A2 + B(1+2)

II. Necrotizing fasciitis
- A. Definite case
  - 1. Necrosis of soft tissues with involvement of the fascia
- PLUS
  - 2. Serious systemic disease, including one or more of the following:
    - a) Death
    - b) Shock (systolic blood pressure <90 mm of Hg).
    - c) Disseminated intravascular coagulopathy
    - d) Failure of organ systems
      - a. respiratory failure
      - b. liver failure
      - c. renal failure
  - 3. Isolation of group A *Streptococcus* from a normally sterile body site
- B. Suspected case
  - 1. 1 + 2 and serologic confirmation of group A streptococcal infection by a 4-fold rise against:
    - a) streptolysin O
    - b) DNase B
  - 2. 1 + 2 and histologic confirmation:
    Gram-positive cocci in a necrotic soft tissue infection

*Streptococcal toxic-shock syndrome (streptococcal TSS) is defined as any group A streptococcal infection associated with the early onset of shock and organ failure. Definitions describing criteria for shock, organ failure, definite cases, and probable cases are included below.
Source: reference 61.

Table 2. Complications of group A streptococcal soft-tissue infection

| Complication | Percentage of Patients |
|---|---|
| Shock | 95 |
| Acute respiratory distress syndrome | 55 |
| Renal impairment | 80 |
| Irreversible | 10 |
| Reversible | 70 |
| Bacteremia | 60 |
| Death | 30 |

Source: reference 1.

occurs in 55% of patients and generally develops after the onset of hypotension (8). Supplemental oxygen, intubation, and mechanical ventilation are necessary in 90% of the patients in whom this syndrome develops. Mortality rates vary from 30% to 70% (1,8,24-26). Morbidity is also high; 13 of 20 patients in one series underwent major surgical procedures, which included fasciotomy, surgical debridement, exploratory laparotomy, intraocular aspiration, amputation, or hysterectomy (8).

### Clinical Isolates

M types 1, 3, 12, and 28 have been the most common isolates from patients with shock and multiorgan failure (8,29). Recently, 80% of strains in Sweden from all types of GAS infection have been M type 1 (S. Holm, pers. comm.). Pyrogenic exotoxin A and/or B was found in most cases of severe infection. In the United States, pyrogenic exotoxin A is most frequently associated with these infections (8,23,29-33), while in Sweden and the United Kingdom, exotoxin B has been most common (12,25). Recently, streptococcal superantigen (SSA), a novel pyrogenic exotoxin, was isolated from an M 3 strain, albeit in small concentrations (34). In addition, mitogenic factor (MF) has been demonstrated in many different M types of GAS (35,36).

### Necrotizing Fasciitis

Necrotizing fasciitis, a deep-seated infection of the subcutaneous tissue that progressively destroys fascia and fat but may spare the skin and muscle, can be caused by GAS, *Clostridium perfringens*, or *C. septicum*. Necrotizing fasciitis caused by mixed organisms such as aerobic gram-negative bacteria, anaerobes, and microaerophilic streptococci may develop in diabetic patients or patients with open wounds contaminated with bowel contents. Though Meleney called infections caused by hemolytic streptococci “streptococcal gangrene” (37), the process has been renamed necrotizing fasciitis. His patients’ infections began at the site of trivial or inapparent trauma. Within 24 hours of the initial lesion—which frequently was only mild erythema—swelling, heat, erythema, and tenderness rapidly developed. During the next 24 to 48 hours, the erythema changed from red to purple and then to blue, and blisters and bullae, which contained clear yellow fluid, appeared. On days 4 and 5, the purple areas became gangrenous. From day 7 to day 10, the line of demarcation became sharply defined, and the dead skin began to separate at the margins or breaks in the center, revealing an extensive necrosis of the subcutaneous tissue. In more severe cases, the process advanced rapidly until several large areas of skin became gangrenous, and the intoxication rendered the patient dull, unresponsive, mentally cloudy, or even delirious. Meleney was the first to advocate aggressive “bear scratch” fasciotomy and debridement. With this treatment, together with irrigation with Dakains solution, the mortality rate dropped to 20% (37).

These older reports of necrotizing fasciitis (6) differ from reports of current necrotizing fasciitis cases associated with streptococcal TSS (8). First, recent cases have mainly occurred in young healthy persons who had no underlying disease but sustained minor trauma to an extremity. Earlier series describe older patients with multiple medical problems (6). Meleney’s cases (reported from China) were probably among young healthy persons who sustained minor trauma, though the major difference between them and present cases is the low mortality rate (20% vs 20% to 60% in streptococcal TSS ) (6,37) before antibiotics were available (37). Analysis of Meleney’s reports also suggests that most of his patients did not have shock or organ failure, nor did they require amputation. In contrast, present cases of necrotizing fasciitis caused by GAS are invariably associated with severe manifestations of systemic illness and high morbidity despite the absence of underlying disease and the use of antibiotics, dialysis, ventilators, intravenous fluids, and improved surgical techniques. In summary, the high mortality rate among current cases of streptococcal necrotizing fasciitis could be due to the emergence of more virulent streptococci (8).

### Streptococcal Myositis

Streptococcal myositis is an extremely uncommon GAS infection. Adams et al. (38) documented only 21 reported cases from 1900 to 1985, and Svane (39) found only four cases in more 20,000 autopsies. Severe pain may be the only early symptom, and swelling and erythema may be the only early physical findings, though muscle compartment syndromes may develop rapidly (8-10,38-41). Distinguishing streptococcal myositis from spontaneous gas gangrene caused by *C. perfringens* or *C. septicum* (42) may be difficult, though crepitus or demonstration of gas in the tissue favors clostridial infection (40). Patients with streptococcal TSS may have both necrotizing fasciitis and myositis (8,38). In published series, the case-fatality rate for necrotizing fasciitis is 20% to 50%, whereas GAS myositis has a fatality rate of 80% to 100% (6). Aggressive surgical debridement is extremely important for establishing a diagnosis and removing devitalized tissue.

### Bacteremia

Streptococcal bacteremia has occurred most commonly in the very young and in the elderly (5). Among children, predisposing factors (other than scarlet fever) include burns, varicella, malignant neoplasm, immunosuppression, and age less than 2 years (5). In patients with scarlet fever, the pharynx is the most common source of GAS. Frequently such patients have complications, such as extension of infection into the sinuses, peritonsillar tissue, or mastoids (septic scarlet fever or scarlet fever anginose); yet documented bacteremia occurs in only 0.3% of febrile patients (43). Among the children with varicella studied by Bullowa and Wischik (43), GAS bacteremia occurred in only approximately 0.5% of patients.

In elderly patients the source of GAS infection is invariably the skin and is associated with cellulitis or erysipelas (5). GAS sepsis in the elderly (mean age, 50 to 60 years) has also been associated with diabetes, peripheral vascular disease, malignancy, and corticosteroid use. Not surprising, mortality rates of 35% to 80% have been described in this patient population. In the past, GAS bacteremia was rare among persons 14 to 40 years of age; puerperal sepsis accounted for most bacteremia in this age group. Recently, intravenous drug abuse has emerged as a leading cause of GAS bacteremia in this age group (5). Martin and Hoiby have comprehensively demonstrated that the prevalence of GAS bacteremia in Norway in the late 1980s increased in all age groups, but the greatest increase (600% to 800%) was in adolescents and young adults (10). Thus, the demographics of invasive streptococcal infections have changed dramatically in the past 4 to 6 years.

### Current Hypotheses Regarding Mechanisms of Shock and Tissue Destruction Caused by Virulent Group A Streptococci

Pyrogenic exotoxins cause fever in humans and animals and also help induce shock by lowering the threshold to exogenous endotoxin (5). Streptococcal pyrogenic exotoxins A and B induce human mononuclear cells to synthesize not only tumor necrosis factor-α (TNFα) (44) but also interleukin-1β (IL-1β) (45) and interleukin-6 (IL-6) (45), suggesting that TNF could mediate the fever, shock, and tissue injury observed in patients with streptococcal TSS (8). Pyrogenic exotoxin C has been associated with mild cases of scarlet fever in the United States (author's observations) and in England (46). The roles of two newly described pyrogenic exotoxins, SSA and MF (see section on "Clinical Isolates"), in streptococcal TSS have not been elucidated.

M protein contributes to invasiveness through its ability to impede phagocytosis of streptococci by human polymorphonuclear leukocytes (47). Conversely, type-specific antibody against the M protein enhances phagocytosis (47). After infection with a particular M type, specific antibody confers resistance to challenge to viable GAS of that M type (47). While M types 1 and 3 strains have accounted for most strains isolated from cases of streptococcal TSS, many other M types, including some nontypable strains, have also been isolated from such cases. M types 1 and 3 are also commonly isolated from asymptomatic carriers, patients with pharyngitis, and patients with mild scarlet fever (7,29).

Could streptococcal TSS be related to the ability of pyrogenic exotoxin or M proteins type 1 or 3 to act as "super antigens" (48)? Data suggest that this exotoxin and a number of staphylococcal toxins (toxic shock syndrome toxin-1 [TSST-1] and staphylococcal enterotoxins A, B, and C) can stimulate T-cell responses through their ability to bind to both the Class II major histocompatibility ability complex of antigen-presenting cells and the $V_\beta$ region of the T-cell receptor (48). The net effect would be to induce T-cell stimulation with production of cytokines capable of mediating shock and tissue injury. Recently, Hackett and Stevens demonstrated that pyrogenic exotoxin A induced both TNFα and TNFβ from mixed cultures of monocytes and lymphocytes (49), supporting the role of lymphokines (TNFβ) in shock associated with strains producing that exotoxin. Kotb et al. (50) have shown that a digest of M protein type 6 can also stimulate T-cell responses by this mechanism; however, the role of specific superantigens in this or any other infectious disease has not been proven. Proof would require demonstration of massive expansion of T-cell subsets bearing a $V_\beta$ repertoire specific for the putative superantigen. However, quantitation of such T-cell subsets in patients with acute streptococcal TSS demonstrated deletion rather than expansion, suggesting that perhaps the life span of the expanded subset was shortened by a process of apoptosis (51). In addition, the subsets deleted were not specific for streptococcal pyrogenic exotoxins A, B, C, or mitogenic factor, suggesting that an as yet undefined superantigen may play a role (51).

Cytokine production by less exotic mechanisms likely contributes as well to the genesis of shock and organ failure. Peptidoglycan, lipoteichoic acid (52), and killed organisms (53,54) are capable of inducing TNFα production by mononuclear cells in vitro

(6,54,55). Exotoxins such as streptolysin O (SLO) are also potent inducers of TNFα and IL-1β. Pyrogenic exotoxin B, a proteinase precursor, has the ability to cleave pre-IL-1β to release preformed IL-1β (56). Finally, SLO and exotoxin A together have additive effects in the induction of IL-1β by human mononuclear cells (49). Whatever the mechanisms, induction of cytokines in vivo is likely the cause of shock, and these two exotoxins, cell wall components, and the like, are potent inducers of TNF and IL-1.

The mere presence of virulence factors, such as M protein or pyrogenic exotoxins, may be less important in streptococcal TSS than the dynamics of their production in vivo. Recently, Cleary et al. proposed a regulon in GAS that controls the expression of a group of virulence genes coding for known virulence factors such as M protein and C5 peptidase (57). When DNA fingerprinting was used, differences were shown between M1 strains isolated from patients with invasive disease and strains from patients with noninvasive GAS infections (58). Finally, genetic information coding for exotoxins A or C may be introduced to strains of GAS by certain bacteriophage; after lysogenic conversion, synthesis of exotoxin A would occur during growth of the streptococcus (31,59,60). Multilocus enzyme electrophoresis demonstrates two patterns that correspond to the M1 and M3 type organisms that produce pyrogenic exotoxin A, a finding that supports epidemiologic studies implicating these strains in invasive GAS infections (33).

The interaction between these microbial virulence factors and an immune or nonimmune host determines the epidemiology, clinical syndrome, and outcome. Since horizontal transmission of GAS in general is well documented, the only explanation for the absence of a high attack rate of invasive infection is significant herd immunity against one or more of the virulence factors responsible for streptococcal TSS. This hypothetical model explains why epidemics have not materialized and why a particular strain of GAS can cause different clinical manifestations in the same community (8,61) (Figure 1).

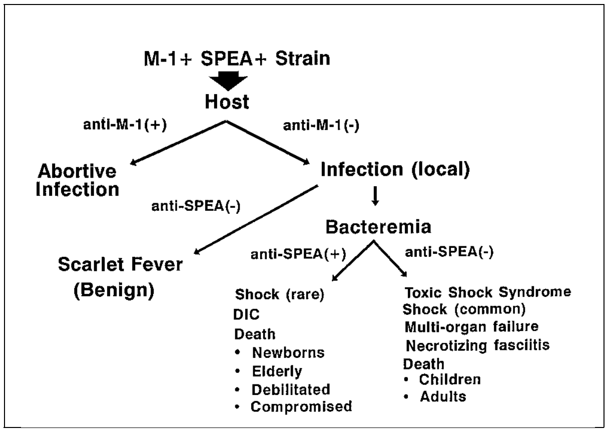

Figure 1. Pathogenesis of scarlet fever, bacteremia, and toxic shock syndrome. M-1$^{+}$ SPEA$^{+}$ = a GAS strain that contains M protein type 1 and streptococcal pyrogenic exotoxin A (SPEA); +anti-M-1 = the presence of antibody to M protein type 1; -anti-M-1 = the absence of antibody to M protein type 1'; anti-SPEA+ = antibody to SPEA; and DIC - disseminated intravascular coagulation.

### Treatment

### Antibiotic Therapy – Cures and Failures with Penicillin

*S. pyogenes* continues to be exquisitely susceptible to ß-lactam antibiotics, and numerous studies have demonstrated the clinical efficacy of penicillin preparations for streptococcal pharyngitis. Similarly, penicillins and cephalosporins have proven efficacy in treating erysipelas, impetigo, and cellulitis, all of which are most frequently caused by *S. pyogenes*. In addition, Wannamaker et al. (6) demonstrated that penicillin therapy prevents the development of rheumatic fever following streptococcal pharyngitis if therapy is begun within 8 to 10 days of the onset of sore throat. Nonetheless, some clinical failures of penicillin treatment of streptococcal infection do occur. Penicillin treatment of *S. pyogenes* has failed to eradicate bacteria from the pharynx of 5% to 20% of patients with documented streptococcal pharyngitis (62-64). In addition, more aggressive GAS infections (such as, necrotizing fasciitis, empyema, burn wound sepsis, subcutaneous gangrene, and myositis) respond less well to penicillin and continue to be associated with high mortality rates and extensive morbidity (6,8,9,12,15,38,65). For example, in a recent report, 25 cases of streptococcal myositis had an overall mortality rate of 85% in spite of penicillin therapy (38). Finally, several studies in experimental infection suggest that penicillin fails when large numbers of organisms are present (66,67).

### The Efficacy of Penicillin, Compared to Clindamycin, In Fulminant Experimental *S. pyogenes* Infection

In a mouse model of myositis caused by *S. pyogenes*, penicillin was ineffective when treatment was delayed ≥2 hours after initiation of infection (67). Survival of erythromycin-treated mice was greater than that of both penicillin-treated mice and untreated controls, but only if treatment was begun within 2 hours. Mice receiving clindamycin, however, had survival rates of 100%, 100%, 80%, and 70%, even if treatment was delayed 0, 2, 6, and 16.5 hours, respectively (67,68).

Eagle suggested that penicillin failed in this type of infection because of the "physiologic state of the organism" (66). This phenomenon has recently been attributed to both in vitro and in vivo inoculum effects (69,70).

### Inoculum Size and the "Physiologic State of the Organism": Differential Expression of Penicillin-Binding Proteins

Penicillin and other ß-lactam antibiotics are most efficacious against rapidly growing bacteria. We hypothesized that large inocula reach the stationary phase of growth sooner than smaller inocula both in vitro and in vivo. That high concentrations of *S. pyogenes* accumulate in deep-seated infection is supported by data from Eagle et al. (66). We compared the penicillin-binding protein patterns from membrane proteins of group A streptococci isolated from different stages of growth, i.e., mid-log phase and stationary phase. Binding of radiolabeled penicillin by all penicillin-binding proteins was decreased in stationary cells; however, PBPs 1 and 4 were undetectable at 36 hours (69). Thus, the loss of certain penicillin-binding proteins during stationary-phase growth in vitro may be responsible for the inoculum effect observed in vivo and may account for the failure of penicillin in treatment of both experimental and human cases of severe streptococcal infection.

### The Greater Efficacy of Clindamycin in Experimental *S. pyogenes* Infections: Mechanisms of Action

The greater efficacy of clindamycin is likely multifactorial: First, its efficacy is not affected by inoculum size or stage of growth (69,71); secondly, clindamycin is a potent suppressor of bacterial toxin synthesis (72,73); third, it facilitates phagocytosis of *S. pyogenes* by inhibiting M-protein synthesis (73); fourth, it suppresses synthesis of penicillin-binding proteins, which, in addition to being targets for penicillin, are also enzymes involved in cell wall synthesis and degradation (71); fifth, clindamycin has a longer postantibiotic effect than ß-lactams such as penicillin; and lastly, clindamycin causes suppression of LPS-induced monocyte synthesis of TNF (74). Thus, clindamycin's efficacy may also be related to its ability to modulate the immune response.

### Other Treatment Measures

Though antibiotic selection is critically important, other measures, such as prompt and aggressive exploration and debridement of suspected deep-seated *S. pyogenes* infection, are mandatory. Frequently, the patient has fever and excruciating pain. Later, systemic toxicity develops, and definite evidence of necrotizing fasciitis and myositis appears. Surgical debridement may be too late at this point. Prompt surgical exploration through a small incision with visualization of muscle and fascia, and timely Gram stain of surgically obtained material may provide an early and definitive etiologic diagnosis. Surgical colleagues should be involved early in such cases, since later in the course surgical intervention may be impossible because of toxicity or because infection has extended to vital areas impossible to debride (i.e., the head and neck, thorax, or abdomen).

Anecdotal reports suggest that hyperbaric oxygen has been used in a handful of patients, though no controlled studies are under way, nor is it clear that this treatment is useful.

Because of intractable hypotension and diffuse capillary leak, massive amounts of intravenous fluids (10 to 20 liters/day) are often necessary. Pressors such as dopamine are used frequently, though no controlled trials have been performed in streptococcal TSS. In patients with intractable hypotension, vasoconstrictors such as epinephrine have been used, but symmetrical gangrene of digits seems to result frequently (author's unpublished observations), often with loss of limb. In these cases it is difficult to determine if symmetrical gangrene is due to pressors, infection, or both.

Neutralization of circulating toxins would be desirable; however, appropriate antibodies are not commercially available in the United States or Europe. Two reports describe the successful use of intravenous gamma globulin in treating streptococcal TSS in two patients (75,76).

In summary, if a wild "flesh-eating strain" has recently emerged, a major epidemic with a high attack rate would normally be expected. Clearly, epidemics of streptococcal infections, including impetigo, pharyngitis, scarlet fever, and rheumatic fever have occurred in the past. However, in the last decade, subsequent to early reports of streptococcal TSS, we have observed that the incidence has remained relatively low. I hypothesize that large outbreaks have not occurred because 1) most of the population probably has immunity to one or more streptococcal virulence factors (6,25); 2) predisposing conditions (e.g., varicella, and use of NSAIDs) are required in a given patient; and 3) only a small percentage of the population may have an inherent predisposition to severe streptococcal infection because of constitutional factors such as HLA Class II antigen type (77,78), B-cell (79), or specific Vβ regions on lymphocytes. This last hypothesis is further supported by the observation that secondary cases of streptococcal TSS, though reported (80), have been rare.

*Dr. Stevens is chief, Infectious Diseases Section, Veterans Affairs Medical Center, Boise, Idaho, and professor of medicine, University of Washington School of Medicine, Seattle. He is a member of CDC's Working*

*Group on Streptococcal Infections and a consultant to the National Institutes of Health and the World Health Organization on Streptococcal Infections. On July 1994, he testified before Congress on Severe Streptococcal Infections and is currently President of the American Lancefield Society.*

### References

1. Stevens DL, Tanner MH, Winship J, Swarts R, Reis KM, Schlievert PM, et al. Reappearance of scarlet fever toxin A among streptococci in the Rocky Mountain West: severe group A streptococcal infections associated with a toxic shock-like syndrome. N Engl J Med 1989; 321:1-7.
2. The Working Group on Severe Streptococcal Infections. Defining the group A streptococcal toxic shock syndrome: rationale and consensus definition. JAMA 1993; 269:390-1.
3. Sennert D. De febribus libri quator. Editio novissima. Cui accessit fasciculus medicamentorum contra pestem. Libri IV. De peste, Pestilentibusque ac Malingis Febribus. Venice: Francisum Baba, 1641.
4. Douglass W. The practical history of a new epidemical eruptive miliary fever, with an Angina Ulcusculosa, which prevailed in Boston, New England in the years 1735 and 1736. Boston: T. Fleet, 1736.
5. Dillon HC. Impetigo contagiosa: suppurative and nonsuppurative complication. Clinical, bacteriologic and epidemiologic characteristics of impetigo. Am J Dis Child 1968; 115:530-41.
6. Wannamaker LW, Rammelkamp CH, Jr., Denny FW, Brink WR, Houser HB, Hahn EO, et al. Prophylaxis of acute rheumatic fever by treatment of the preceding streptococcal infection with various amounts of depot penicillin. Am J Med 1951; 10:673-95.
7. Weaver GH. Scarlet Fever. In: Abt IA, ed., Pediatrics. Philadelphia: W.B. Saunders Co., 1925:298-362.
8. Stevens DL. Invasive group A streptococcus infections. Clin Infect Dis 1992; 14:2-13.
9. Kohler W, Gerlach D, Knoll H. Streptococcal outbreaks and erythrogenic toxin type A. Zbl Bakt Hyg 1987; 266:104-15.
10. Martin PR, Hoiby EA. Streptococcal serogroup A epidemic in Norway 1987-1988. Scand J Infect Dis 1990; 22:421-9.
11. Holm S. Fatal group A streptococcal infections. Presented at the 89th Conference of the American Society for Microbiology, New Orleans, LA,1989.
12. Wheeler MC, Roe MH, Kaplan EL, Schlievert PM, Todd JK. Outbreak of group A streptococcus septicemia in children: clinical, epidemiologic, and microbiological correlates. JAMA 1991; 266:533-7.
13. Gaworzewska ET, Coleman G. Correspondence: group A streptococcal infections and a toxic shock-like syndrome. N Engl J Med 1989; 321:1546.
14. Schwartz B, Facklam R, Breiman R. The changing epidemiology of group A streptococcal infections in the U.S.: association with changes in serotype. Presented at the 30th Interscience Conference on Antimicrobial Agents and Chemotherapy, Atlanta, GA, 1990; Abstract 88.
15. Bartter T, Dascal A, Carroll K, Curley FJ. "Toxic strep syndrome": manifestation of group A streptococcal infection. Arch Intern Med 1988; 148:1421-4.
16. Hribalova V. *Streptococcus pyogenes* and the toxic shock syndrome. Ann Intern Med 1988; 108:772.
17. Greenberg RN, Willoughby BG, Kennedy DJ, Otto TJ, McMillian R, Bloomster TG. Hypocalcemia and "toxic" syndrome associated with streptococcal fasciitis. South Med J 1983; 76:916-8.
18. Jackson MA, Olson LC, Burry VF. Pediatric group A streptococcal (GAS) disease with multi-organ dysfunction. Presented at the 30th Interscience Conference on Antimicrobial Agents and Chemotherapy, Atlanta, GA, 1990; Abstract 195.
19. Thomas JC, Carr SJ, Fujioka K, Waterman SH. Community-acquired group A streptococcal deaths in Los Angeles County. J Infect Dis 1989; 160:1086-7.
20. Francis J, Warren RE. *Streptococcus pyogenes* bacteraemia in Cambridge: a review of 67 episodes. Q J Med 1988; 256:603-13.
21. Barnham M. Invasive streptococcal infections in the era before the acquired immune deficiency syndrome: a 10 years' compilation of patients with streptococcal bacteraemia in North Yorkshire. J Infect Dis 1989; 18:231-48.
22. Braunstein H. Characteristics of group A streptococcal bacteremia in patients at the San Bernardino County Medical Center. Rev Infect Dis 1991; 13:8-11.
23. Schwartz B, Facklam RR, Brieman RF. Changing epidemiology of group A streptococcal infection in the USA. Lancet 1990; 336:1167-71.
24. Holm SE, Norrby A, Bergholm AM, Norgren M. Aspects of pathogenesis of serious group A streptococcal infections in Sweden, 1988-1989. J Infect Dis 1992; 166:31-7.
25. Stegmayr B, Bjorck S, Holm S, Nisell J, Rydvall A, Settergren B. Septic shock induced by group A streptococcal infections: clinical and therapeutic aspects. Scand J Infect Dis 1992; 24:589-97.
26. Demers B, Simor AE, Vellend H, Schlievert PM, Byrne S, Jamieson F, et al. Severe invasive group A streptococcal infections in Ontario, Canada: 1987-1991. Clin Infect Dis 1993; 16:792-800.
27. Auerbach SB, Schwartz B, Facklam RR, Breiman R, Jarvis WR. Outbreak of invasive group A streptococcal (GAS) disease in a nursing home. Presented at the 30th Interscience Conference on Antimicrobial Agents and Chemotherapy, Atlanta, GA, 1990; Abstract 171.
28. Hohenboken JJ, Anderson F, Kaplan EL. Invasive group A streptococcal (GAS) serotype M-1 outbreak in a long-term care facility (LTCF) with mortality. Presented at the 34th Interscience Conference on Antimicrobial Agents and Chemotherapy, Orlando, FL, 1994; Abstract J189.
29. Johnson DR, Stevens DL, Kaplan EL. Epidemiologic analysis of group A streptococcal serotypes associated with severe systemic infections, rheumatic fever, or uncomplicated pharyngitis. J Infect Dis 1992; 166:374-82.
30. Belani K, Schlievert P, Kaplan E, Ferrieri P. Association of exotoxin-producing group A streptococci and severe disease in children. Pediatr Infect Dis J 1991; 10:351-4.

31. Hauser AR, Goshorn SC, Kaplan E, Stevens DL, Schlievert PM. Molecular analysis of the streptococcal pyrogenic exotoxins. Presented at the Third International American Society for Microbiology Conference on Streptococcal Genetics. Minneapolis, MN, 1990.
32. Hauser AR, Stevens DL, Kaplan EL, Schlievert PM. Molecular analysis of pyrogenic exotoxins from *Streptococcus pyogenes* isolates associated with toxic shock-like syndrome. J Clin Microbiol 1991; 29:1562-7.
33. Musser JM, Hauser AR, Kim MH, Schlievert PM, Nelson K, Selander RK. *Streptococcus pyogenes* causing toxic-shock-like syndrome and other invasive diseases: clonal diversity and pyrogenic exotoxin expression. Proc Natl Acad Sci USA 1991; 88:2668-72.
34. Mollick JA, Miller GG, Musser JM, Cook RG, Grossman D, Rich RR. A novel superantigen isolated from pathogenic strains of *Streptococcus pyogenes* with aminoterminal homology to staphylococcal enterotoxins B and C. J Clin Invest 1993; 92:710-9.
35. Iwasaki M, Igarashi H, Hinuma Y, Yutsudo T. Cloning, characterization and overexpression of a *Streptococcus pyogenes* gene encoding a new type of mitogenic factor. FEBS Lett 1993; 331:187-92.
36. Norrby-Teglund A, Newton D, Kotb M, Holm SE, Norgren M. Superantigenic properties of the group A streptococcal exotoxin SpeF (MF). Infect Immun 1994; 62:5227-33.
37. Meleney FL. Hemolytic *Streptococcus* gangrene. Arch Surg 1924; 9:317-64.
38. Adams EM, Gudmundsson S, Yocum DE, Haselby RC, Craig WA, Sundstrom WR. Streptococcal myositis. Arch Intern Med 1985; 145:1020-3.
39. Svane S. Peracute spontaneous streptococcal myositis: a report on 2 fatal cases with review of literature. Acta Chir Scand 1971; 137:155-63.
40. Yoder EL, Mendez J, Khatib R. Spontaneous gangrenous myositis induced by *Streptococcus pyogenes*: case report and review of the literature. Rev Infect Dis 1987; 9:382-5.
41. Nather A, Wong FY, Balasubramaniam P, Pang M. Streptococcal necrotizing myositis — a rare entity: a report of two cases. Clin Orthop 1987; 215:206-11.
42. Stevens DL, Musher DM, Watson DA, Eddy H, Hamill RJ, Gyorkey F, Rosen H, et al. Spontaneous, nontraumatic gangrene due to *Clostridium septicum*. Rev Infect Dis 1990; 12:286-96.
43. Bullowa JGM, Wischik S. Complications of varicella. I: their occurrence among 2,534 patients. Am J Dis Child 1935;49: 923-6.
44. Fast DJ, Schlievert PM, Nelson RD. Toxic shock syndrome-associated staphylococcal and streptococcal pyrogenic toxins are potent inducers of tumor necrosis factor production. Infect Immun 1989; 57:291-4.
45. Hackett SP, Schlievert PM, Stevens DL. Cytokine production by human mononuclear cells in response to streptococcal exotoxins. Clin Res 1991; 39:189A.
46. Hallas G. The production of pyrogenic exotoxins by group A streptococci. J Hyg (Camb) 1985; 95:47-7.
47. Lancefield RC. Current knowledge of type specific M antigens of group A streptococci. J Immunol 1962; 89:307-13.
48. Mollick JA, Rich RR. Characterization of a superantigen from a pathogenic strain of *Streptococcus pyogenes*. Clin Res 1991; 39:213A.
49. Hackett SP, Stevens DL. Streptococcal toxic shock syndrome: synthesis of tumor necrosis factor and interleukin-1 by monocytes stimulated with pyrogenic exotoxin A and streptolysin O. J Infect Dis 1992; 165:879-85.
50. Kotb M, Tomai M, Majumdar G, Walker J, Beachey EH. Cellular and biochemical responses of human T lymphocytes stimulated with streptococcal M protein. Presented at the 11th Lancefield International Symposium on Streptococcal Diseases, Siena, Italy, 1990; Abstract L77.
51. Watanabe-Ohnishi R, Low DE, McGeer A, Stevens DL, Schlievert PM, Newton D, et al. Selective depletion of Vβ-bearing T cells in patients with severe invasive group A streptococcal infections and streptococcal toxic shock syndrome. J Infect Dis 1995; 171:74-84.
52. Stevens DL, Bryant AE, Hackett SP. Gram-positive shock. Curr Opin Infect Dis 1992; 5:355-63.
53. Hackett S, Ferretti J, Stevens D. Cytokine induction by viable group A streptococci: suppression by streptolysin O. Presented at the 93rd Conference of the American Society for Microbiology, Las Vegas, NV, 1994; Abstract B-249.
54. Muller-Alouf H, Alouf JE, Gerlach D, Ozegowski JH, Fitting C, Cavaillon JM. Comparative study of cytokine release by human peripheral blood mononuclear cells stimulated with *Streptococcus pyogenes* superantigenic erythrogenic toxins, heat-killed streptococci and lipopolysaccharide. Infect Immun 1994; 62:4915-21.
55. Hackett SP, Stevens DL. Superantigens associated with staphylococcal and streptococcal toxic shock syndromes are potent inducers of tumor necrosis factor beta synthesis. J Infect Dis 1993; 168:232-5.
56. Kappur V, Majesky MW, Li LL, Black RA, Musser JM. Cleavage of Interleukin 1B (IL-1B) precursor to produce active IL-1B by a conserved extracellular cysteine protease from *Streptococcus pyogenes*. Proc Natl Acad Sci USA 1993; 90:7676-80.
57. Cleary R, Chen C, Lapenta D, Bormann N, Heath D, Haanes E. A virulence regulon in Streptococcus pyogenes. Presented at the Third International American Society for Microbiology Conference on Streptococcal Genetics, Minneapolis, MN, 1990; Abstract 19.
58. Cleary PP, Kaplan EL, Handley JP, Wlazlo A, Kim MH, Hauser AR, et al. Clonal basis for resurgence of serious *Streptococcus pyogenes* disease in the 1980s. Lancet 1992; 339:518-21.
59. Nida SK, Ferretti JJ. Phage influence on the synthesis of extracellular toxins in group A streptococci. Infect Immun 1982; 36:745-50.
60. Johnson LP, Tomai MA, Schlievert PM. Bacteriophage involvement in group A streptococcal pyrogenic exotoxin A production. J Bacteriol 1986; 166:623-7.
61. Stevens DL. Invasive group A streptococcal infections: the past, present and future. Pediatr Infect Dis J 1994; 13:561-6.
62. Kim KS, Kaplan EL. Association of penicillin tolerance with failure to eradicate group A streptococci from patients with pharyngitis. J Pediatr 1985; 107:681-4.

63. Gatanaduy AS, Kaplan EL, Huwe BB, McKay C, Wannamaker LW. Failure of penicillin to eradicate group A streptococci during an outbreak of pharyngitis. Lancet 1980; 2:498-502.
64. Brook I. Role of beta-lactamase-producing bacteria in the failure of penicillin to eradicate group A streptococci. Pediatr Infect Dis 1985; 4:491-5.
65. Kohler W. Streptococcal toxic shock syndrome. Zbl Bakt 1990; 272:257-64.
66. Eagle H. Experimental approach to the problem of treatment failure with penicillin. I. Group A streptococcal infection in mice. Am J Med 1952; 13:389-9.
67. Stevens DL, Gibbons AE, Bergstrom R, Winn V. The Eagle effect revisited: efficacy of clindamycin, erythromycin, and penicillin in the treatment of streptococcal myositis. J Infect Dis 1988; 158:23-8.
68. Stevens DL, Bryant AE, Yan S. Invasive group A streptococcal infection: new concepts in antibiotic treatment. Int J Antimicrob Agents 1994; 4:297-301.
69. Stevens DL, Yan S, Bryant AE. Penicillin-binding protein expression at different growth stages determines penicillin efficacy in vitro and in vivo: an explanation for the inoculum effect. J Infect Dis 1993; 167:1401-5.
70. Yan S, Mendelman PM, Stevens DL. The in vitro antibacterial activity of ceftriaxone against *Streptococcus pyogenes* is unrelated to penicillin-binding protein 4. FEMS Microbiol Lett 1993; 110:313-18.
71. Yan S, Bohach GA, Stevens DL. Persistent acylation of high-molecular weight penicillin-binding proteins by penicillin induces the post-antibiotic effect in *Streptococcus pyogenes*. J Infect Dis 1994; 170:609-14.
72. Stevens DL, Maier KA, Mitten JE. Effect of antibiotics on toxin production and viability of *Clostridium perfringens*. Antimicrob Agents Chemother 1987; 31:213-8.
73. Gemmell CG, Peterson PK, Schmeling D, Kim Y, Mathews J, Wannamaker L, et al. Potentiation of opsonization and phagocytosis of *Streptococcus pyogenes* following growth in the presence of clindamycin. J Clin Invest 1981; 67:1249-56.
74. Stevens DL, Bryant AE, Hackett SP. Antibiotic effects on bacterial viability, toxin production and host response. Clin Infect Dis 1995;20(Suppl 2):S154-7.
75. Barry W, Hudgins L, Donta ST, Pesanti EL. Intravenous immunoglobulin therapy for Toxic shock syndrome. JAMA 1992; 267(24):3315-6.
76. Yong JM. Letter. Lancet 1994; 343:1427.
77. Greenberg LJ, Gray ED, Yunis E. Association of HL-A5 and immune responsiveness in vitro to streptococcal antigens. J Exp Med 1975; 141:934-43.
78. Weinstein L, Barza M. Gas gangrene. N Engl J Med 1972; 289:1129.
79. Zabriskie JB, Lavenchy D, Williams RCJ, et al. Rheumatic-fever associated B-cell alloantigens as identified by monoclonal antibodies. Arthritis Rheum 1985; 28:1047-51.
80. Schwartz B, Elliot JA, Butler JC, Simon PA, Jameson BL, Welch GE, et al. Clusters of invasive group A streptococcal infections in family, hospital, and nursing home settings. Clin Infect Dis 1992; 15:277-84.
